# A diagnosis-based clinical decision rule for spinal pain part 2: review of the literature

**DOI:** 10.1186/1746-1340-16-7

**Published:** 2008-08-11

**Authors:** Donald R Murphy, Eric L Hurwitz, Craig F Nelson

**Affiliations:** 1 Rhode Island Spine Center, 600 Pawtucket Avenue, Pawtucket, RI, 02860, USA; 2 Department of Community Health, Warren Alpert Medical School of Brown University, USA; 3 Research Department, New York Chiropractic College, USA; 4 Department of Public Health Sciences and Epidemiology, John A. Burns School of Medicine, University of Hawaii at Mânoa, Honolulu, Hawaii, 96822, USA; 5 American Specialty Health, San Diego, CA, USA

## Abstract

**Background:**

Spinal pain is a common and often disabling problem. The research on various treatments for spinal pain has, for the most part, suggested that while several interventions have demonstrated mild to moderate short-term benefit, no single treatment has a major impact on either pain or disability. There is great need for more accurate diagnosis in patients with spinal pain. In a previous paper, the theoretical model of a diagnosis-based clinical decision rule was presented. The approach is designed to provide the clinician with a strategy for arriving at a specific working diagnosis from which treatment decisions can be made. It is based on three questions of diagnosis. In the current paper, the literature on the reliability and validity of the assessment procedures that are included in the diagnosis-based clinical decision rule is presented.

**Methods:**

The databases of Medline, Cinahl, Embase and MANTIS were searched for studies that evaluated the reliability and validity of clinic-based diagnostic procedures for patients with spinal pain that have relevance for questions 2 (which investigates characteristics of the pain source) and 3 (which investigates perpetuating factors of the pain experience). In addition, the reference list of identified papers and authors' libraries were searched.

**Results:**

A total of 1769 articles were retrieved, of which 138 were deemed relevant. Fifty-one studies related to reliability and 76 related to validity. One study evaluated both reliability and validity.

**Conclusion:**

Regarding some aspects of the DBCDR, there are a number of studies that allow the clinician to have a reasonable degree of confidence in his or her findings. This is particularly true for centralization signs, neurodynamic signs and psychological perpetuating factors. There are other aspects of the DBCDR in which a lesser degree of confidence is warranted, and in which further research is needed.

## Background

Accurate diagnosis or classification of patients with spinal pain has been identified as a research priority [1]. We presented in Part 1 the theoretical model of an approach to diagnosis in patients with spinal pain [2]. This approach incorporated the various factors that have been found, or in some cases theorized, to be of importance in the generation and perpetuation of neck or back pain into an organized scheme upon which a management strategy can be based. The authors termed this approach a diagnosis-based clinical decision rule (DBCDR). The DBCDR is not a clinical prediction rule. It is an attempt to identify aspects of the clinical picture in each patient that are relevant to the perpetuation of pain and disability so that these factors can be addressed with interventions designed to improve them. The purpose of this paper is to review the literature on the methods involved in the DBCDR regarding reliability and validity and to identify those areas in which the literature is currently lacking.

### The Three Essential Questions of Diagnosis

The DBCDR is based on what the authors refer to as the 3 essential questions of diagnosis [2]. The answers to these questions supply the clinician with the most important information that is required to develop an individualized diagnosis from which a management strategy can be derived. The 3 questions are:

#### 1. Are the symptoms with which the patient is presenting reflective of a visceral disorder or a serious or potentially life-threatening disease?

In seeking the answer to this question, history and examination and, when indicated, special tests, are used to detect or raise the level of suspicion for the presence of pathological disorders for which spinal pain may be the first or only symptom. Some examples are gastrointestinal or genitourinary disorders, fracture, infection and malignancy. Potentially serious or life-threatening conditions are sometimes referred to as "red flags" [3].

#### 2. From where is the patient's pain arising?

In seeking the answer to this question, four signs are searched for: (1) centralization signs, (2) segmental pain provocation signs, (3) neurodynamic signs, and (4) muscle palpation signs.

#### 3. What has gone wrong with this person as a whole that would cause the pain experience to develop and persist?

In seeking the answer to this question, perpetuating factors are searched for: (1) dynamic instability (impaired motor control), (2) central pain hypersensitivity, (3) oculomotor dysfunction (in cervical trauma patients), (4) fear, (5) catastrophizing, (6) passive coping, and (7) depression. These latter psychological factors are sometimes referred to as "yellow flags" [4].

**Figure 1:**
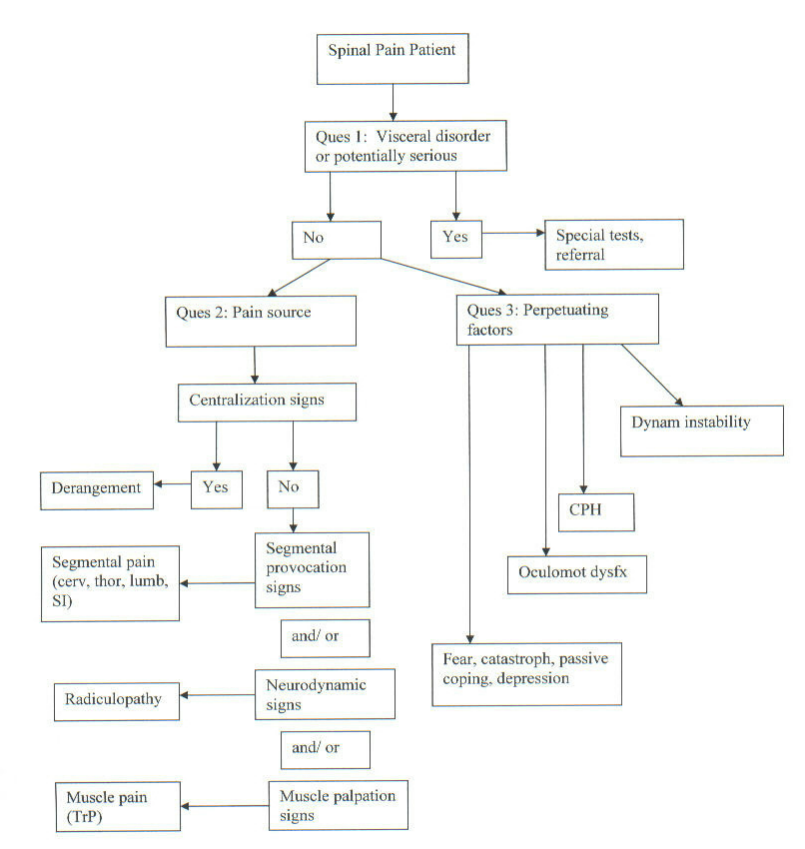
**Diagnostic algorithm for the application of the DBCDR**.

**Figure 2:**
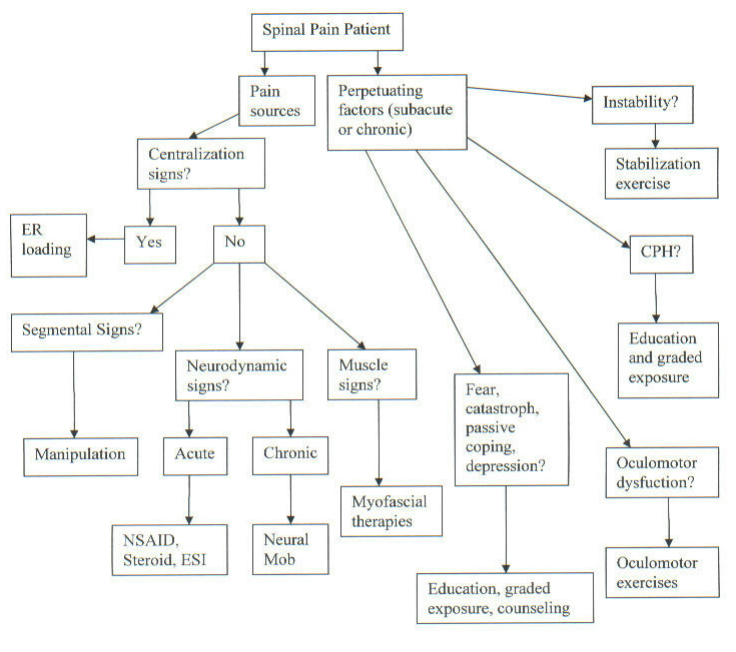
Management algorithm for the application of the DBCDR.

An algorithm illustrating the diagnostic strategy of the DBCDR is presented in figure 1. The recommended management strategy based on the DBCDR is presented in figure 2.

The purpose of this paper is to review the literature on the reliability and validity of the detection of the individual diagnostic factors included in the DBCDR, and to present the evidence as it currently exists, for the various aspects of this approach.

## Methods

### Literature search and selection

The following databases were searched up to December 22, 2006: Medline, Cinahl, Embase and MANTIS. Searches of the authors' own libraries were also conducted. Finally, citation searches of relevant articles and texts were conducted manually. The following search terms were used:

Diagnosis AND "low back pain"

Diagnosis AND "neck pain"

Diagnosis AND "low back pain" AND palpation

Diagnosis AND "neck pain" AND palpation

Diagnosis AND "low back pain" AND McKenzie

Diagnosis AND "neck pain" AND McKenzie

Diagnosis AND "low back pain" AND neurodynamics

Diagnosis AND "neck pain" AND neurodynamics

Diagnosis AND "low back pain" AND radiculopathy

Diagnosis AND "neck pain" AND radiculopathy

Diagnosis AND "low back pain" AND trigger points

Diagnosis AND "neck pain" AND trigger points

Diagnosis AND "low back pain" AND muscle

Diagnosis AND "neck pain" AND muscle

Diagnosis AND "low back pain" AND instability

Diagnosis AND "neck pain" AND instability

Diagnosis AND "low back pain" AND "motor control"

Diagnosis AND "neck pain" AND "motor control"

Diagnosis AND "low back pain" AND "central sensitization"

Diagnosis AND "low back pain" AND "central pain hypersensitivity"

Diagnosis AND "neck pain" AND "central sensitization"

Diagnosis AND "neck pain" AND "central pain hypersensitivity"

Diagnosis AND "neck pain" AND oculomotor

Diagnosis AND "low back pain" AND fear

Diagnosis AND "neck pain" AND fear

Diagnosis AND "low back pain" AND catastrophizing

Diagnosis AND "neck pain" AND catastrophizing

Diagnosis AND "low back pain" AND coping

Diagnosis AND "neck pain" AND coping

Diagnosis AND "low back pain" AND depression

Diagnosis AND "neck pain" AND depression

Studies were included if they were in English and provided original, statistically analyzed data regarding the reliability and validity of clinic-based diagnostic procedures used for the identification of relevant factors in the causation or perpetuation of spinal pain. Included studies had to contain data on the assessment of patients with cervical or lumbar pain, including headache related to the cervical spine and spine-related upper or lower extremity pain. Non-English language studies were excluded, as were studies that did not present data on reliability and validity. The search focused on diagnostic procedures that are potentially useful in answering the second or third question of diagnosis. Studies that were potentially useful in answering question 1 were not considered for the purpose of this paper. Diagnostic studies that require special equipment not typically found in the clinic (such as MRI) or that require a laboratory (such as blood tests) were excluded because the purpose of the study was to evaluate clinic-based means by which the DBCDR may be applied. It is recognized that imaging or laboratory tests are often useful in the diagnosis of spinal pain, but the presentation of these procedures was beyond the scope of this paper. In cases in which systematic reviews of the literature were found, the individual studies included in the reviews were not reviewed separately, unless this was necessary to clarify information that was not readily apparent from the systematic review.

Each study was reviewed by two authors (DRM and CFN) and deemed relevant or irrelevant. A study was considered relevant if the information contained in the study indicated that it met the above inclusion/exclusion criteria.

## Results

The search strategy identified 1769 articles, and of these, 138 were deemed relevant. Additional files 1 and 2 provide a breakdown of the number of studies in each area of consideration. Additional files 3 and 4 present the data from those studies that met the inclusion criteria. We have divided the presentation of the literature into those studies that apply to patients with neck pain and those that relate to patients with low back pain (LBP).

### Neck Pain

#### Question 1. Are the symptoms with which the patient is presenting reflective of a visceral disorder or a serious or potentially life-threatening disease?

A detailed review of the literature related to this question is beyond the scope of this paper. However, in general, history, focusing on the presence of symptoms such as GI distress, fever or previous history of cancer, and examination, focusing on vital signs, abdominal examination and examination of peripheral pulses, are useful in raising the level of suspicion as to the presence of a visceral disorder or a serious or potentially life-threatening disease [5]. Imaging and/or special tests such as sedimentation rate can be utilized for further confirmation [5]. Details can be found elsewhere [5-7].

#### Question 2. From where is the patient's pain arising?

##### Centralization signs

Centralization signs are detected through methods originally developed by McKenzie [8,9]. The examination procedure involves moving the spine to end range in various directions and monitoring the mechanical and symptomatic response to these movements.

###### Reliability

Clare, et al [10] used 2 physical therapists trained in the McKenzie method to examine 25 patients with cervical pain. They found good inter-examiner reliability (IER) (*kappa*, [*k*] = 0.63 and 93% agreement) for the assessment procedure.

###### Validity

No studies were identified that have addressed the validity of centralization signs in the cervical spine.

##### Segmental pain provocation signs

A number of studies have examined segmental mobility assessment and have generally found poor IER [11-16] and validity [17]. Other studies have examined procedures designed to identify segmental pain (as opposed to mobility impairment).

###### Reliability

Hubka and Phelan [18] assessed the IER of palpation for tenderness between 2 practitioners in 30 patients with unilateral neck pain. They found good IER (*k *= 0.68). Jull, et al [19] assessed IER of segmental palpation using 7 examiners and 40 subjects with or without neck pain and headache. The criteria for a positive test were based on resistance to joint movement and pain provocation in response to palpation. Kappa values indicated excellent to perfect IER (*k *= 0.78–1.00) in 6 instances, fair to good (*k *= 0.45–0.65) in 14 instances and poor (*k *= 0.25–0.34) in 5 instances. They point out that, in the instances of poor agreement, the raw data indicated that the examiners had agreed on 13 of 14 decisions. But the calculations of *k *were vulnerable because 12 of the 13 agreements were in the same cell of agreed negative finding. Marcus, et al [20] used 4 physical therapists to examine 72 headache patients and 24 controls. The therapists examined all subjects for "cervical synovial joint abnormalities" in the same manner as described in the study by Jull, et al [19]. They found good IER (*k *= 0.63) between examiners. McPartland and Goodridge [21] assessed IER of "TART" exam, described as segmental palpation that focused on three parameters: tissue texture change, restriction of vertebral motion and zygapophyseal (z) joint tenderness. They found the IER of examination that considered all three parameters was poor (*k *= 0.35 for asymptomatic subjects, *k *= 0.34 for symptomatic subjects). But for the parameter of tenderness alone, IER improved (*k *= 0.529). Van Suijlekom, et al [22] used 2 neurologists to examine 24 headache patients and found IER for segmental palpation to be slight to fair (*k *= 0.14 to 0.37). However, the palpation method was poorly described in this study. Also, it is not known as to whether the difference between the findings of this study and those of the other studies reported here relate to the fact that the "negative" IER studies used neurologists, whereas the "positive" IER study used chiropractors or physical therapists. Cleland, et al [23] used 2 examiners and 22 subjects and found highly variable IER between 2 physical therapists for palpation for pain provocation, with *k *ranging from -.52 to .90, depending on the segment involved. They speculated that this high variability related to the clinicians not agreeing on the segmental level being examined, as opposed to lack of agreement on the findings.

###### Validity

Jull, et al [24] used diagnostic blocks to identify the presence and location of symptomatic z joints in 20 patients with cervical related pain. The patients were examined by a manipulative physiotherapist who also attempted to identify the presence and location of symptomatic z joints. The definition of a symptomatic joint as determined by palpation was based on abnormal "end feel", increased resistance to motion and reproduction of pain. They found that the SE and SP were both 1.00. That is, the examiner was able to identify 100% of the symptomatic segments as well as all of the subjects whose pain was not abolished by diagnostic block. This study used single, rather than double blind, diagnostic blocks. Regardless, as will be discussed below, the use of diagnostic blocks as a Gold Standard for the presence of z joint pain has been questioned [25]. Treleaven, et al [26] assessed 12 patients with postconcussion headache with segmental palpation. The method of palpation was the same as that used by Jull, et al [24]. They found complete agreement between the examiner and independent report of the patient as to which segments were painful and almost complete agreement as to which segment was most painful. Sandmark and Nisell [27], calculated the SE, SP and PPV and negative predictive value (NPV) of segmental palpation in the cervical spine relative to reported neck pain. They found these values to be 0.82, 0.79, 0.62 and 0.91 respectively. Lord, et al [28], used a double blind anesthetic block to determine the prevalence of pain arising from the C2-3 z joint in patients with the complaint of chronic headache after cervical trauma. These authors demonstrated that the prevalence of C2-3 z-joint pain was 53%, and the only sign that was associated with these patients was tenderness to palpation over the C2-3 z joint. They calculated that palpation had SE of 0.85, a positive likelihood ratio (PLR) of 1.7 and a negative likelihood ratio (NLR) of 0.3. The precise method of palpation was not described. Zito, et al [29] using the palpation method found to be reliable by Jull et al [19] found a significantly higher incidence (p < 0.05) of hypomobile and painful z joints in the upper cervical spine of patients classified according to the International Headache Society criteria as having cervicogenic headache compared to those classified as having migraine with aura. King, et al [30] used "controlled, diagnostic blocks" as a Gold Standard against which segmental palpation that was described as being similar to that of Jull, et al [24]. They found the SE to be 0.88, SP to be 0.39 and PLR to be 1.3. Again, using diagnostic block as a Gold Standard may be questionable [25], leaving open the issue of what should be the Gold Standard for segmental palpation signs. Further work in the area of establishing a true Gold Standard for the identification of zygapophyseal joint pain may be needed before definitive statements regarding the presence or absence of pain from this structure can be made.

##### Neurodynamic signs

###### Reliability

The standard neurodynamic test in the cervical spine is the brachial plexus tension test (also known as the upper limb tension test [31]). Wainner, et al [32] found good to excellent IER of this test (*k *= 0.76 to 0.81). They also found good to excellent IER of several historical questions of patients with documented cervical radiculopathy (*k *= 0.53 to .082). They found varying IER of neurologic exam findings, but good to excellent IER of Spurling's test (which they described as bending the seated patient's head toward the side of symptoms, rotating and extending slightly, and applying downward pressure), the cervical distraction test and Valsalva's maneuver. The kappa values for these tests ranged from 0.60 to 0.88.

###### Validity

Wainner, et al [32] provide data on the SE, SP PLR and NLR of a variety of historical factors and examination procedures. They found that the cluster of 4 tests – Spurling's test, the upper limb tension test, the cervical distraction test and limited rotation toward the side of symptoms secondary to pain – carried the greatest diagnostic accuracy as compared to the Gold Standard of electromyography. When 3 of these tests were positive, there was a 65% probability of the presence of cervical radiculopathy the SE and SP were 0.39 and 0.94, respectively and a PLR of 6.1. When all 4 tests were positive, there was a 90% probability of the presence of cervical radiculopathy. The SE and SP were 0.24 and 0.99 respectively and the PLR was 30.3.

Shah and Rajshekhar [33] also used Spurling's test, the description of which was the same as that in the Wainner, et al study [32], and found it to be useful in identifying "soft disc prolapse" as opposed to "hard disc" (i.e., osteophyte). They calculated the SE and SP to be 0.90 and 1.00, respectively compared to the Gold Standard of operative findings. The PPV was calculated to be 1.00 and the NPV to be 0.71. In patients treated non-surgically, they used MRI as the Gold Standard and calculated the SE and SP to be 0.90 and 0.93, respectively. The PPV was calculated to be 0.90 and the NPV to be 0.93.

##### Muscle palpation signs

###### Reliability

Marcus, et al, in the same study cited above [20] found good to perfect IER of TrP palpation in the cervical spine (*k *= 0.74), head (*k *= 0.81) and shoulder (*k *= 1.00). van Suijlekom, et al [22] in the study cited above, found variable IER (*k *= 0.0 – 1.00) of TrP palpation in patients with headache. As was the case with segmental palpation, the method of TrP examination was poorly described. Gerwin, et al [34] performed 2 different experiments to assess IER. In the first, 4 examiners assessed 20 different muscles on each of 25 patients with various symptom presentations. They used a general observer-agreement statistic called the "*S*_av_", which they defined as "a generalized version of the Cohen's kappa which reports pairwise judge agreement corrected for chance agreement." They found poor IER (*S*_av _= 0.0–1.0). They then repeated the study after spending a 3-hour session in which the examiners discussed positive findings and palpation techniques. They found good to excellent IER (*S*_av _= 0.65 – .95) after the training session. Sciotti, et al [35] found good IER (Generalizability coefficient = 0.83–0.92) between 2 examiners looking for latent trigger points (TrPs) in the upper trapezius muscle. However, the subjects were asymptomatic. On the other hand, Lew, et al [36] found poor IER for TrP palpation in the upper trapezius, although the subjects in that study were also asymptomatic.

###### Validity

The validity of muscle palpation signs is unknown, largely due to lack of an appropriate Gold or reference standard.

### 3. What has gone wrong with this person as a whole that would cause the pain experience to develop and persist?

As was discussed in the earlier paper describing the DBCDR [2], this third question attempts to identify those factors that may be placing the patient at risk of developing persistent or recurrent spinal pain, or, in the case of chronic patients, have contributed to the establishment of the chronic or recurrent problem. There are a number of factors that have been suggested to be of importance in the perpetuation of chronic spinal pain, although research investigating this area is ongoing.

#### Dynamic instability (impaired motor control)

##### Reliability

In the cervical spine, the Craniocervical Flexion (CF) test [37,38] is designed to detect decreased activity in the deep cervical flexor muscles and hyperactivity in the sternocleidomastoid muscles. It is thought that, as the deep cervical flexors are important for stability of the intersegmental joints of the cervical spine, this imbalance in muscle activation compromises cervical spine stability [37]. The CF test measures the motor control capacity of the deep cervical flexors. Jull, et al [38] found good IER (ICC = 0.81 to 0.93) in 50 asymptomatic subjects; Chiu, et al [39] found good IER (*k *= 0.72) in 10 asymptomatic subjects.

Recently, 3 studies [23,40,41] have demonstrated IER of a test that uses a similar positioning but, rather than using a pressure cuff, involves practitioner observation of the ability of patients to maintain a position of slight upper cervical flexion in the supine position. Cleland, et al [23] used 2 examiners and 22 subjects and found moderate IER (ICC = 0.57). Harris, et al [40] used 2 examiners and 40 subjects and found moderate IER (ICC = 0.67); Olson, et al [41], using an almost identical test as Harris, et al [40], found excellent IER (*k *= 0.83 to 0.88) between 2 examiners in 27 subjects without neck pain.

##### Validity

Treleavan, et al [26] compared 12 patients with postconcussion headache with asymptomatic controls using the CF test. They found a significant (*p *= 0.02) decrease in the duration of time that the test position could be held in patients compared to controls. Jull, et al [38] compared 15 patients with cervicogenic headache and compared them with 15 controls. They found significantly (*p *< 0.001) poorer performance on the CF test in the patients compared to controls. Jull, et al [42] compared patients with neck pain after whiplash, patients with insidious onset neck pain and normal controls in the performance of the CF test. They found significantly poorer performance (*p *< 0.05) in both neck pain groups than in controls. There was no difference between the post-whiplash patients and the insidious onset patients. Falla, et al [43] used the CF test and electromyography (EMG) to demonstrate reduced activity in the deep cervical flexor muscles in patients with chronic neck pain compared to controls. There was also a trend toward increased activity in the sternocleidomastoid and scalene muscles in patients compared to controls. With regard to increased activity in the sternocleidomastoid muscle during the performance of the CF test, this replicated the findings of Jull [44].

#### Central Pain Hypersensitivity (CPH)

As will be discussed below, there is good evidence that the presence of nonorganic signs is reflective of increased pain perception. [45]

##### Reliability

Sobel, et al [46] developed nonorganic signs for patients with neck pain and found excellent to perfect (*k *= 0.80 to 1.00) IER in 26 patients.

##### Validity

The validity of cervical nonorganic signs is unknown.

Imaging modalities like functional MRI and SPECT have promise in the diagnosis of CPH [47,48]; however, it is not clear as to whether these are viable tools for common use.

#### Oculomotor dysfunction

Oculomotor dysfunction has been found in patients with chronic neck pain after whiplash [49] as well as in patients with chronic tension type headache [50]. Gimse, et al [51] compared 26 patients with chronic (average 4.7 years) neck pain after whiplash and who had complaints of visual problems or vertigo and compared them with 26 matched controls. They found significantly (*p *< 0.001) poorer performance on tests of oculomotor function in the whiplash group. Tjell, et al [52] compared 160 chronic (a minimum of 6 months) neck pain patients whose pain was attributed to whiplash with 122 patients with either non-traumatic neck pain, dizziness related to the cervical spine and fibromyalgia. Using the same method of measurement of oculomotor function used by Gimse, et al [51], they found significantly (*p *< 0.05 to *p *< 0.0001) poorer performance on tests of oculomotor function in the whiplash patients compared to the other groups. There currently are no simple tests for oculomotor reflex function that are practical for the typical clinical setting. However, Heikkilla and Wenngren [53] found significant correlation between the finding of poor performance on oculomotor tests and on a test for head repositioning accuracy, which can be measured in the clinic using Revel's test [54].

Revel, et al [54] originally demonstrated that patients with chronic neck pain had significantly (*p *< 0.01) poorer repositioning accuracy compared to a group of 30 asymptomatic controls. Loudon, et al [55] also found significantly (*p *< 0.05) poorer repositioning accuracy in patients with chronic neck pain after whiplash compared to healthy controls; however, the small sample size (11 subjects in each group) makes interpretation problematic. Heikkilla and Wenngren [53] found significantly greater error in patients (n = 27) with chronic neck pain after whiplash compared to 39 controls. As was stated earlier, Heikklla and Wenngren [53] found close correlation (*p *= 0.007) between poor head repositioning accuracy and dysfunction of oculomotor reflexes.

Treleaven, et al [56] also found close correlation between head repositioning accuracy (which they termed "joint position error") and oculomotor function. They calculated the SE and SP of using head repositioning accuracy to predict oculomotor dysfunction to be 0.60 and 0.54, respectively and the PPV to be 0.88.

#### Fear and Catastrophizing

Several instruments have been used to measure fear and catastrophizing. Regarding fear, the best studied are the Fear-Avoidance Beliefs Questionnaire [57], the Tampa Scale for Kinesiophobia [58] and the Fear-Avoidance Pain Scale [59].

In patients with neck pain, measures of fear have been found to predict future chronicity in both non-traumatic neck pain [60] and neck pain after whiplash [61,62], although there is some conflicting evidence [63].

### Passive coping

The Vanderbilt Pain Management Inventory has been demonstrated to be a reliable and valid measure of passive coping [64] and this measure has been found to predict slower recovery from whiplash injury [65].

#### Depression

The Center for Epidemiologic Studies Depression (CES-D) Scale [66] has been found to have good internal consistency and responsiveness to change over time as well as validity as compared to clinical criteria, self-report criteria, need for services and association with life events [67]. Depressive symptoms as measured by the CES-D have been found to contribute to slower recovery from whiplash injury [65].

### Low Back Pain

#### Question 1. Are the symptoms with which the patient is presenting reflective of a visceral disorder or a serious or potentially life-threatening disease?

As stated earlier, a detailed review of the literature related to this question is beyond the scope of this paper. The discussion of this question in the neck pain section of the paper applies to this section as well.

#### Question 2. From where is the patient's pain arising?

##### Centralization signs

###### Reliability

Early studies [68,69] failed to demonstrated adequate IER of the McKenzie assessment in the lumbar spine. For example, Riddle and Rothstein [68] looked at 363 patients with LBP and used 49 physical therapists at 8 different clinics and found poor IER (*k *= 0.26) of the classification systems of McKenzie. Postgraduate training in the system did not improve IER. However, these studies have been criticized on the grounds that minimally trained therapists were used, the study failed to consider the classification of patients into subsyndromes and, in the case of Kilby, et al [69], the protocol included elements that are not a standard part of the McKenzie system [10]. More recent studies have attempted to improve upon the methodology of these earlier studies. Werneke, et al [70] used 5 physical therapists who assessed 289 patients with LBP or neck pain and found IER that ranged from *k *= 0.917 to 1.0. Fritz, et al [71] used 40 physical therapists in practice and 40 physical therapy students and had them watch a video of 12 examinations using the McKenzie method. They found IER coefficients ranging from *k *= 0.763 to 0.823. Razmjou, et al [72] used 2 trained McKenzie therapists and 45 patients with acute, subacute or chronic LBP and found good IER (*k *= 0.70). Kilpikosk, et al [73] looked at 39 patients with low back pain examined by 2 physical therapists trained in the McKenzie method. They found good agreement for the presence of the centralization sign (*k *= 0.7) and excellent agreement for direction preference (*k *= 0.9). Clare, et al [10] found perfect IER (*k *= 1.0) between 2 examiners in 25 patients with LBP.

###### Validity

Donelson, et al [74] found that the McKenzie assessment differentiated discogenic from nondiscogenic pain (*p *< 0.001), using discogram as the Gold Standard. Young, et al [75] used the Donelson, et al [74] data and calculated the sensitivity (SE) and specificity (SP) to be 0.94 (95% confidence interval [CI] 0.82, 0.99) and 0.52 (95% CI 0.34, 0.69), respectively. Young, et al [75], using their own original data, calculated the SE and SP of centralization signs to be 0.47 and 1.00, respectively, also using discography as the Gold Standard. They also found that pain upon arising from a sitting position was associated with disc pain (*p *= .017). This historical factor may therefore be useful in identifying the "centralizer", though as will be noted below, pain when arising from sitting is also associated with segmental pain provocation signs in the sacroiliac (SI) area. Laslett, et al [76] also used discogram as the Gold Standard and calculated the SE, SP, and positive likelihood ratio (PLR) and negative likelihood ratio (NLR) for centralization signs to be 40%, 94%, 6.9 and 0.63 respectively. They also used the Roland Morris Disability questionnaire to measure disability and the Distress Risk Assessment Method to measure distress, and found these factors altered the SE, SP and PPV. In the presence of severe disability, these values were 46%, 80%, 3.2 and 0.63 respectively and in the presence of severe distress they were 45%, 89%, 4.1 and 0.61 respectively.

It is pointed out by Long, et al [77], that it is not necessary to assume a particular pain generating tissue when using the McKenzie assessment as a means of making treatment decisions. In their study, clinical decisions were made regarding exercise direction based on the findings of the end range loading examination. One group of patients were given exercise maneuvers in the direction of centralization of symptoms, another was given exercises in the direction opposite that of centralization, and a third group was given exercises that did not consider any specific direction. They found significantly greater improvement (*p *< 0.001) in outcome in the patients who were given exercises in the direction of centralization, suggesting that the McKenzie evaluation in the lumbar spine allows clinicians to make treatment decisions that are of ultimate benefit to patients. This may be a more important measure of "validity" than the identification of a certain pain generating tissue (e.g., using a prognostic criterion as a reference standard for the assessment method).

Centralization signs have also been found to be predictive of long term outcome. Werneke and Hart [78] found that discriminating between patients who exhibit centralization signs from those who do not allows for prediction of pain, disability and return to work at 1 year. In a separate study, Werneke and Hart [79] compared classification according to centralization signs with classification according to the Quebec Task Force (QTF) criteria [80]. They found that examination for centralization signs had greater predictive validity for pain and disability at discharge from care than the QTF criteria. Werneke and Hart have also found that assessing centralization signs over the period of multiple visits allows for more accurate discrimination than a single assessment [81].

##### Segmental pain provocation signs

###### Reliability – lumbar

Similar to what was found for the cervical spine, palpation for movement restriction in the lumbar spine has not been shown to be reliable, though palpation for pain has. Keating, et al [82] used 3 chiropractors who examined 25 asymptomatic subjects and 21 patients with low back pain. They found marginal to good IER of palpation for pain provocation over bony structures (*k *= 0.19 to 0.48) and soft tissues (*k *= 0.10 to 0.59). The strongest IER was found for the L4-5 and L5-S1 segments. Maher and Adams [83] used 2 examiners to assess 90 subjects with low back pain, allowing each examiner to use whatever palpation method he or she chose. The examiners assessed each patient for pain and stiffness. They found that, while the IER of palpation for stiffness was low (intraclass correlation coefficient [ICC] = 0.03–0.37) the IER for pain was good (ICC = 0.67–0.72). Strender, et al [84] used 2 medical physicians and 2 physical therapists to evaluate 71 patients with low back pain. They found moderate agreement (*k *= 0.40) for palpation for tenderness. Lundberg, et al [85] used 2 examiners to assess 609 female subjects for segmental mobility and pain provocation through palpation. They found good IER (*k *= 0.67 – 0.71) for this assessment.

Seffinger, et al [86] systematically reviewed the literature regarding the IER of palpatory diagnosis in both neck and back pain. They concluded that palpatory procedures for pain provocation generally have acceptable IER (*k *= 0.40 or greater) and that 64% of studies looking at pain provocation found acceptable IER.

###### Reliability – Sacroiliac area

With regard to the SI area, the earliest study of IER was that of Potter and Rothstein [87]. They did not use the kappa statistic, but they found that tests that attempt to determine movement abnormality had poor reliability (less than 70% agreement) but the 2 tests that relied on patient response had agreement of 70–90%. Carmichael [88] also found poor IER (*k *= 0.314) of an SI test that assessed for mobility. Freburger and Riddle [89] found poor reliability (*k *= 0.18) of the measurement of SI joint position using handheld calipers. Robinson, et al [90] evaluated the reliability of various pain and SI joint dysfunction tests. The palpation test for joint play showed very poor reliability (*k *= -0.06). Other pain provocation tests demonstrated moderate to good reliability (k = 0.43–0.84). When clustered results of three to five pain provocation tests were used there was also good reliability (*k *= 0.51–0.75). A study by Vincent-Smith and Gibbons [91] evaluated the IER and intra-examiner reliability of the standing flexion test for SI joint dysfunction. Intra-examiner reliability was moderate (*k *= 0.46) while IER was very poor (*k *= 0.052).

Tong, et al [92] tested the hypothesis that combining the test results of various measures of SI joint dysfunction would yield greater reliability than individual tests. They established three methods to be evaluated; Method 1: using the test result with the highest IER; Method 2: requiring at least one test result to be abnormal for the variable to be abnormal, and; Method 3: requiring all test results to be abnormal for the variable to be abnormal. Kappa scores were 0.47, 0.08, and 0.32 using Method 1 for the sacral position, innominate bone position, and side of sacroiliac joint dysfunction, respectively. For Method 2 the values were 0.09, 0.4, and 0.16. For Method 3 the values were 0.16, 0.1, and -0.33.

Laslett and Williams [93] used 2 examiners to evaluate 51 patients using 6 tests designed to identify a painful SI joint. They found moderate to high IER (*k *= 0.69 to 0.82), of several tests. Dreyfuss, et al [94] found moderate IER (*k *= 0.61 to 0.64) for 3 SI pain provocation tests. Kokmeyer, et al [95] found good IER (*k *= 0.70) of a cluster of 5 SI pain provocation tests. Studies that have evaluated tests of SI mobility have generally found poor IER [96].

###### Validity – lumbar

Young, et al [75] found a correlation between abolishment of pain with facet joint blocks and the absence of a historical report of pain when standing from a sitting position. Revel, et al [97] found that the following characteristics were associated with patients whose pain was relieved by 75% or more with facet joint blocks: age over 65, pain not exacerbated by coughing, pain not worsened by hyperextension, pain not worsened by forward flexion, pain not worsened by rising from forward flexion, pain not worsened by extension-rotation and pain well relieved with recumbency. Similar findings have been found by other authors [98,99]. Laslett, et al [100] found that these criteria had low SE (< 0.17), though they did have high SP (0.90). Laslett, et al [101] found that 4 or more out of the following 7 signs carried a SE of 1.00 and SP of 0.87 as compared to single facet joint blocks: Age ≥ 50, symptoms best walking, symptoms best sitting, onset pain is paraspinal, Modified Somatic Perception Questionnaire score > 13, positive extension/rotation test, and absence of centralization signs. So, as will be seen in the SI joint area, ruling out centralization signs is necessary to increase the diagnostic yield in identifying segmental pain provocation signs.

###### Validity – SI joint area

In the SI joint area, Broadhurst and Bond [102] compared 3 pain provocation tests with anesthetic block and found the SE of single tests ranged from 0.77 to 0.87. The SP of each test was 1.00. Slipman, et al [103] used a cluster of pain provocation tests and used the criteria of at least 3 "positive" tests in 50 consecutive patients with LBP. They compared this examination with the Gold Standard of single anesthetic blocks. They estimated the PPV of the examination to be 60%. van der Wurff, et al [104] assessed 140 patients with chronic LBP with a cluster of 5 pain provocation maneuvers for the SI joint. This cluster was the same as that used in the study by Kokmeyer, et al [95] that had found good IER. They considered that 3 out of the 5 tests being pain-producing constituted a "positive" test. They compared this regimen with the Gold Standard of double anesthetic blocks. They calculated the SE of the regimen as 0.85 (95% CI, 0.72–0.99) the SP as 0.79 (95% CI, 0.65–0.93), and the PPV and NPV as 0.77 (95% CI, 0.62–0.92) and 0.87 (95% CI, 0.74–0.99), respectively. The PLR was 4.02 (95% CI, 2.04–7.89); the NLR was 0.19 (95% CI, 0.07–0.47). Laslett, et al [105] used these same SI provocation tests and compared these to single anesthetic block. They added to the Gold Standard criteria the reproduction of concordant pain upon infiltration, followed by 80% or more reduction of pain as a result of injection. They found that the presence of 3 positive tests carried a SE of 0.94, a SP of 0.78, a PPV of 0.68, and a NPV of 0.96. Young, et al [75] also found significant (*p *< .001) association between the presence of 3 or more positive pain provocation tests for the SI and positive SI injection and also found positive association between positive SI injection and the following historical factors: pain when arising from a sitting position (*p *= .02), pain being unilateral (*p *= .05) and the absence of midline pain (*p *= .05). They also noted that patients with positive SI injection rarely had pain superior to the L5 level.

Importantly, Laslett, et al [106] found that performing SI provocation maneuvers in the context of the end range loading exam for centralization signs (see below) increases the diagnostic yield of the SI tests. The SP of the SI provocation tests rose from 0.78 to 0.87 and the PLR rose from 4.16 to 6.97.

Slipman, et al [107] compared radionuclide imaging to the Gold Standard of single SI joint block and found this test to have high SP (100%) but very low SE (12.9%).

##### Neurodynamic signs

###### Reliability

The standard neurodynamic tests in the lumbar spine are the Straight Leg Raise (SLR), Femoral Nerve Stretch test (FNST – also sometimes referred to as the Prone Knee Bend [108]) and the Slump test. Clinicians will often include Bragard's test (adding ankle dorsiflexion to the SLR) and the Well Leg Raise (WLR) test (eliciting pain on the affected side by performing a SLR on the contralateral limb) to serve as sensitizing and differentiating maneuvers for the purpose of increasing the specificity of the examination for lower lumbar nerve root pain [109].

Hunt, et al [110] assessed the IER of the SLR using 2 teams of examiners, each team consisting of one physician and one physical therapist. They found fair IER (*k *= 0.54 for left leg, 0.48 for right leg) but this study used asymptomatic subjects and measured SLR using a goniometer. Vroomen, et al [111], used a neurologist and a neurology resident to assess 338 patients with "sciatica". They calculated the IER of a variety historical factors and clinical tests in patients with suspected lumbar radiculopathy. For the standard SLR, they found good IER (*k *= 0.68) when the interpretation of the test findings included the production of "typically dermatomal pain". The *k *values for the Bragard's and WLR tests were 0.66 and 0.70, respectively. When historical and examination factors were taken into consideration regarding arriving at a diagnosis of nerve root pain, the *k *value was 0.66. The historical factors with the greatest IER were increased pain with coughing/sneezing/straining (*k *= 0.64), increased pain with walking (*k *= 0.56), coldness in the lower extremity (*k *= 0.56), urinary incontinence (*k *= 0.79) and previous back pain episodes (*k *= 0.67).

McCombe, et al [112] used 2 surgeons to assess 50 patients and found fair agreement for the FNST (*k *= 0.3–0.5). Philip, et al [113] used 6 pairs of physiotherapists to examine 93 patients using the Slump test. They found good to perfect IER (*k *= 0.72 to 1.00). Gabbe, et al [114] used a physiotherapist and a research student to assess 15 asymptomatic volunteers using the slump test and found excellent reliability (ICC = 0.92, 95% CI 0.77, 0.97).

###### Validity

Vroomen, et al [115] found that SLR was not predictive of the presence of herniated disc on MRI. They did not assess WLR or Bragard's test. They did note that the historical factors of a dermatomal distribution of pain, increase in pain on coughing, sneezing, or straining, paroxysmal pain, and predominant leg pain were predictive. Using MRI as a "Gold Standard" may be questionable because of the potential for false positive findings [116]. Lurie [117] reviewed the literature on diagnostic tests for LBP and found that the SLR has generally been found to have high SE (0.78 to 0.97) but low SP (0.10 to 0.52) in identifying patients with disc herniation. The opposite is found for WLR test, which has been found to have low SE (0.22 to 0.52) and high SP (0.85 to 1.0). He does note, however that "much of the literature is limited by methodological flaws". Many clinicians feel that the combination of the SLR and WLR, along with Bragard's test and other "localizing" and "sensitizing" maneuvers improves the SE and SP of the examination for pain of neural origin [109]. This has not been specifically evaluated.

The validity of the FNST has not been well studied [117].

Stankovic, et al [118] found those patients with the complaint of LBP and/or leg pain whose imaging revealed a herniated disc were more likely to have distal pain in the lower extremity on the performance of the Slump test, although the difference was not statistically significant (*p *< 0.017). No values with regard to SE, SP and PPV and NPV were calculated.

##### Myofascial Signs

###### Reliability

Nice, et al [119] used 12 examiners to assess 50 patients with LBP for trigger points, using the standard criteria of the presence of a "taut band" and localized "nodule", the presence of a "twitch response" and the reproduction of familiar pain. They found IER to be poor (*k *= 0.29 to 0.38). Njoo and Van der Does [120] also found poor IER when considering all of the standard criteria of TrP presence. However, when considering only tenderness to palpation, particularly when combined with the identification of concordant pain on the part of the patient, IER increased greatly (*k *> 0.5). Hsieh, et al [121] used 1 "expert" DC with many years of experience with TrP palpation, 2 DC's with 15 years of practice experience but not with extensive experience with TrP palpation, and several chiropractic and psychiatry residents. They provided all clinicians with 3 2-hour lectures and 3 2-hour hands-on sessions as training in TrP palpation, and compared the agreement between the expert and the others for the presence of taut band, local twitch response and referred pain. They found generally poor IER, concluding that even with experienced clinicians, short term training in TrP palpation is not enough to provide IER.

It would appear that if the examiner places greatest emphasis on tenderness to palpation and reproduction of concordant pain, and less emphasis on the presence of a taut band and a twitch response, the IER of muscle palpation signs will be enhanced. Also, Simons has pointed out [122] that those studies using untrained and/or inexperienced examiners have generally found poor IER, whereas those using trained and experienced examiners have generally found favorable IER in TrP examination, indicating the importance of examiners having appropriate training and experience with muscle palpation signs.

###### Validity

As with the cervical spine, the validity of myofascial signs in the lumbar spine is unknown due to the absence of a Gold Standard for the identification of myofascial pain.

### 3. What has gone wrong with this person as a whole that would cause the pain experience to develop and persist?

#### Dynamic instability (impaired motor control)

##### Reliability

There are 3 tests that have been proposed to identify the presence of dynamic instability in the lumbar spine, and for which there are data on IER. One is the Segmental Instability test [123], which Hicks, et al [123] found to have excellent (*k *= .87) IER between 3 pairs of examiners in 63 subjects. This study [123] found the Standing Flexion test to have moderate IER (*k *= .69). The Hip Extension test [124], was found by Murphy, et al [124] to have good (*k *= 0.72 to 0.76) IER between 2 examiners in 42 subjects.

##### Reliability – pelvis

The Active Straight Leg Raise (ASLR) test [125] is designed to assess dynamic stability in the pelvis. IER of the ASLR has not been evaluated, however, Mens, et al [126] test-retest reliability over the space of one week to be high (Pearson's correlation coefficient = 0.87; ICC = 0.83) in a study of pregnant women.

##### Validity – lumbar

The only validity study that was found was that of Abbott, et al [127]. This study assessed manual examination using intervertebral motion tests. They compared this with a reference standard using flexion-extension radiographs. They provided SE, SP and PPV data, however, no data were presented with regard to the IER of the manual examination procedures, making interpretation of the validity data difficult.

##### Validity – pelvis

Mens, et al [126] compared the ASLR test with the Posterior Pelvic Pain Provocation (PPPP) test, a test with good reliability and validity [126] for the identification of painful SI joints. Using the PPPP test as the Gold Standard, they found the ASLR test to have a SE of 0.87 and a SP of 0.94. In another study, Mens, et al [128] compared the ASLR test to the Quebec Back Pain Disability Scale in 200 pregnant patients with posterior pelvic pain. They found a high correlation between the 2 tests (*r *= 0.70). O'Sullivan et al [129] found evidence of altered activity in the diaphragm and the pelvic floor muscles, both of which are thought to play important roles in motor control of the trunk, in patients with a positive ASLR as compared to those with a normal test. No actual measures of pelvic motor control were performed, however.

#### Central Pain Hypersensitivity (CPH)

##### Reliability

There is some evidence for the IER of Waddell's nonorganic signs, although this evidence is inconsistent [45].

##### Validity

Fishbain, et al [45] reviewed the literature on the use of Waddell's nonorganic signs and found consistent evidence that they are associated with decreased functional performance, poor treatment outcome and increased pain perception. Whether the relationship between the presence of these signs and increased pain perception means that these signs are an indication of CPH specifically is unknown. However, until further investigation is undertaken, it appears that these signs may be a useful means to identify increased pain perception that may be related to CPH.

#### Fear and Catastrophizing

The Fear-Avoidance Beliefs Questionnaire [57], the Tampa Scale for Kinesiophobia [58] and the Fear-Avoidance Pain Scale [59] have been demonstrated to be predictive of present LBP as well as future progression of chronicity [130-134]. Regarding catastrophizing, the Pain Catastrophizing Scale [132,134] has been found to be useful.

These measures have been found to predict decreased physical performance and perceived disability in patients with acute LBP [132], current pain intensity and disability in patients with chronic LBP [130], and reduction in disability after treatment [134].

#### Passive Coping

The Guarding scale of the Chronic Pain Coping Inventory [131] and the Coping Strategies Questionnaire [135] have been found to be predictive, in part, of chronicity in patients with LBP.

#### Depression

The Beck Depression Inventory (BDI) has been used for a number of years in patients with spinal pain, and has been demonstrated to have good utility in identifying significant depressive symptoms in LBP patients [136]. Walsh, et al [137] found that a Mental Component Summary cutoff score of 35 on the SF-36 instrument carried a SE of 0.80 and a SP of 0.90 compared to the Gold Standard of the CES-D. Low scores on the SF-36 Mental Health Index are associated both cross-sectionally and longitudinally with low-back pain and disability [138] suggesting that psychological distress may be both a predictor and consequence of spinal pain. The Depression Anxiety Stress Scales (DASS) have been found to have good internal consistency and reliability, and to compare favorably with the BDI [139], although this study was not performed with spinal pain patients. Haggman, et al [140] used receiver operating characteristic curves to compare the administration of a 2 question screening ("During the past month, have you often been bothered by feeling down, depressed, or hopeless?" and "During the past month, have you often been bothered by little interest or pleasure in doing things?") with the DASS. They found the screening questions accurately predicted DASS scores (Area Under the Curve [AUC] values of 0.77 to 0.81). The PLR reached as high as 5.40 and the NLRs as low as 0.18. Whether this 2-question screening is useful for research purposes is unclear.

As was stated in Part 1, there is significant overlap and interaction between fear, catastrophizing, passive coping and depression [141,142]. Thus, from a clinical standpoint, it may be only necessary to measure 1 or 2 of these constructs in spinal pain patients, rather than having to measure all, however research is needed to determine this for certain.

## Summary

In a previous paper the authors presented the conceptual model of a novel approach to the diagnosis and treatment of patients with spinal pain. The specific components of the diagnostic model were described and the decision making process based on the diagnostic approach were discussed. In this paper, the evidence as it currently exists for the reliability and validity of the components of the diagnostic model is presented. Future research will be conducted to investigate those questions that remain unanswered with regard to the ability of clinicians to arrive at a specific diagnosis in patients with spinal pain on which they can base a targeted treatment approach.

## Competing interests

The authors declare that they have no competing interests.

## Authors' contributions

DRM conceived of the idea of the diagnosis-based clinical decision rule, led the literature search and review process, and was the principle author of the manuscript. ELH was responsible for help with design and presentation of the systematic review, assisted with the conceptualization of the presented research strategy and contributed to the writing of the manuscript. CFN was responsible for performing literature searches and reviews and contributed to the writing of the manuscript. All authors read and approved the final manuscript.

## Supplementary Material

Additional file 1 Table 1. Number of studies identified that address factors related to question number 2. Click here for file

Additional file 2 Table 2. Number of studies identified that address factors related to question number 3. Click here for file

Additional file 3 Table 3. Findings from studies related to question 2. Click here for file

Additional file 4 Table 4. Findings from studies related to question 3. Click here for file
