# Supplementary material for: A diagnosis-based clinical decision rule for spinal pain part 2: review of the literature
Source: Chiropr Osteopat. 2008 Aug 11;16:7. doi: 10.1186/1746-1340-16-7 (PMC2538525; doi:10.1186/1746-1340-16-7)
Supplement: Additional file 1 — Table 1. Number of studies identified that address factors related to question number 2. [file 1746-1340-16-7-S1.doc]

Table 1. Number of studies identified that address factors related to question number 2.

| Factor | Total studies | Reliability | Validity |
| --- | --- | --- | --- |
| Centralization signs | 15 | 7 | 8 |
| Segmental signs | 38 | 20 | 18 |
| Neurodynamic signs | 10 | 5 | 5 |
| Muscle signs | 8 | 8 | 0 |
