# Supplementary material for: A diagnosis-based clinical decision rule for spinal pain part 2: review of the literature
Source: Chiropr Osteopat. 2008 Aug 11;16:7. doi: 10.1186/1746-1340-16-7 (PMC2538525; doi:10.1186/1746-1340-16-7)
Supplement: Additional file 2 — Table 2. Number of studies identified that address factors related to question number 3. [file 1746-1340-16-7-S2.doc]

Table 2. Number of studies identified that address factors related to question number 3.

| Factor | Total studies | Reliability | Validity |
| --- | --- | --- | --- |
| Dynamic instability | 18 | 9 | 9 |
| CPH | 4^a^ | 2^a^ | 3 ^a^ |
| Oculomotor dysfunction | 6 | 0^b^ | 6 |
| Fear/ catastrophizing | 14 | 0^b^ | 14 |
| Passive coping | 5 | 0^b^ | 5 |
| Depression | 8 | 0^b^ | 8 |

CPH – central pain hypersensitivity; ^a^ - includes one systematic review; ^b^Assessment does not require examiner interpretation
