# Supplementary material for: A diagnosis-based clinical decision rule for spinal pain part 2: review of the literature
Source: Chiropr Osteopat. 2008 Aug 11;16:7. doi: 10.1186/1746-1340-16-7 (PMC2538525; doi:10.1186/1746-1340-16-7)
Supplement: Additional file 3 — Table 3. Findings from studies related to question 2. [file 1746-1340-16-7-S3.doc]

Table 3. Findings from studies related to question 2.

| Test | Study | Reliability | Validity |
| --- | --- | --- | --- |
| **Centralization Signs** |  |  |  |
| Cervical (Reliability) |  |  |  |
| McKenzie | Clare [10] | *k*=0.63 |  |
| Cervical (Validity) | None |  |  |
| Lumbar (Reliability) |  |  |  |
|  | Riddle [68] | *k*=0.26 |  |
|  | Werneke [70] | *k*=0.917 – 1.0 |  |
|  | Fritz [71] | *k*=0.763 – 0.823 |  |
|  | Razmjou [72] | *k*=0.70 |  |
|  | Kilpikoski [73] | *k*=0.70  (centralization)  *k=*0.90  (directional preference) |  |
|  | Claire [10] | (*k*=1.0) |  |
| Lumbar (Validity) |  |  |  |
|  | Donelson [74] |  | Significant (p<0.001) correlation with discogram |
|  | Young [75] |  | SE = 0.47  SP = 1.00 |
|  | Laslett [76] |  | SE = 40%  SP = 94%  PLR = 6.9  NLR = 0.63 |
|  | Long [77] |  | Improved outcomes using direction of centralization to direct treatment  (p<.001) |
|  | Werneke [78] |  | Improved prediction of pain (p<.002), disability (p<.001), return to work (p<.001) |
|  | Werneke [79] |  | Improved prediction of pain at discharge (p<0.001) and disability at discharge (p<0.001) and at one year (SE=0.68, SP=0.82, PPV=0.41, NPV=0.94) |
| **Segmental Pain Provocation Signs** |  |  |  |
| Cervical (reliability) | Hubka [18] | *k*=0.68 |  |
|  | Jull [19] | (*k* = 0.78-1.00) in 6 instances  (*k* = 0.45-0.65) in14 instances  (*k* = 0.25-0.34) in 5 instances |  |
|  | Marcus [20] | *k*=0.63 |  |
|  | McPartland [21] | Asymptomatic subjects  *(k*=0.35)  Symptomatic subjects  *(k*=0.34*)*  Tenderness  *(k*=0.53*)* |  |
|  | Van Suijlekom [22] | *(k*=0.14-0.37*)* |  |
|  | Cleland [23] | *(k*=-0.52-.90*)* |  |
| Lumbar (Reliability) |  |  |  |
|  | Keating [82] | Bony structures  *(k*=0.19-0.48*)*  Soft tissues  *(k*=0.10-0.59*)* |  |
|  | Maher [83] | Stiffness  *(ICC*=0.03-0.37*)*  Pain  *(ICC*=0.67-0.72*)* |  |
|  | Strender [84] | Tenderness  *(k*=0.40*)* |  |
|  | Lundberg [85] | Pain  *(k*=0.67=0.71*)* |  |
|  | Seffinger [86] | *(k*=0.40*)* |  |
|  |  |  |  |
| SI (Reliability) |  |  |  |
|  | Potter and Rothstein [87] | Mobility  (<70% agreement)  Patient response  (70-90% agreement) |  |
|  | Carmichael [88] | Mobility  (*k*=0.314) |  |
|  | Freburger and Riddle [89] | Position  (*k*=0.18) |  |
|  | Laslett [93] | *(k*=0.69-0.82*)* |  |
|  | Dreyfuss [94] | *(k*=0.15–0.64) |  |
|  | Vander Wurff [96] | Systematic Review |  |
|  | Kokmeyer [95] | *(k*=0.70*)* |  |
|  | Robinson [90] | Palpation  *(k*=-0.06*)*  Provocation  *(k=*0.43-0.84*)*  Cluster  *(k=*0.6-0.75*)* |  |
|  | Tong [92] | Method I  *(k=*0.08-0.47*)*  Method II  *(k=*0.09-0.4*)*  Method III  *(k=-*0.33-0.16*)* |  |
|  | Vincent-Smith [91] | Interexaminer  *(k=*0.52*)*  Intraexaminer  *(k=*0.46*)* |  |
| Cervical (Validity) |  |  |  |
|  | Jull [24] |  | Diagnostic blocks  SE = 1.00  SP = 1.00 |
|  | Treleaven [26] |  | Complete agreement examiner and independent report |
|  | Sandmark [27] |  | Segmental Palpation  SE = 0.82  SP = 0.79  PPV = 0.62  NPV = 0.91 |
|  | Lord [28] |  | SE = 0.85  PLR = 1.7  NLR = 0.3 |
|  | Zito [29] |  | Significant (p<0.05) correlation between segmental palpation findings and presence of cervicogenic headache patients |
|  | King [30] |  | SE = 0.88  SP = 0.39  PLF = 1.3 |
| Lumbar (Validity) |  |  |  |
| Absence of pain on sit-to-stand | Young [75] |  | Significant (p=0.008) correlation with facet block |
| Revel criteria: Age 65; pain not exacerbated by coughing; pain not worsened by hyperextension; pain not worsened by forward flexion; pain not worsened by rising from forward flexion; pain not worsened by extension/rotation; pain relieved by recumbency | Revel [97] |  | Significant (P < 0.0001) correlation with facet block |
| Pain arising from sitting position | Young [75] |  | Significant (p=.02) correlation with facet block |
| Unilateral pain | Young [75] |  | Significant (p=.05) correlation with facet block |
| Absence of midline pain | Young [75] |  | Significant (p=.05) correlation with facet block |
| Revel criteria | Laslett [100] |  | SE = 0.17  SP = 0.90 |
| Four of more of: Age≥50; symptoms best walking; symptoms best sitting; onset pain is paraspinal; Modified Somatic Perception Questionnaire score >13; positive extension/rotation test; absence of centralization signs | Laslett [101] |  | SE = 1.00  SP = 0.87 |
|  |  |  |  |
| SI joint (Validity) |  |  |  |
|  | Dreyfuss [94] |  | SE = 0.36 – 0.93  SP = 0.15 – 0.64  LR = 0.7 – 1.3 |
|  | Broadhurst [102] |  | SE = 0.77 - 0.87  SP = 1.00 |
|  | Slipman [103] |  | PPV = 0.60 |
|  | Van der Wurff [104] |  | SE = 0.85  SP = 0.79  PPV = 0.77  NPV = 0.87  PLR = 4.02  NLR = 0.19 |
|  | Kokmeyer [95] |  | SE = 0.85  SP = 0.79  PPV = 0.77  NPV = 0.87  PLR = 4.02  NLR = 0.19 |
|  | Laslett [105] |  | SE = 0.94  SP = 0.78  PPV = 0.68  NPV = 0.96 |
|  | Laslett [106] |  | SP = 0.87  PLR = 6.97 |
|  | Slipman [107] |  | SE = 1.0  SE = 0.13 |
|  |  |  |  |
| Neurodynamic Signs |  |  |  |
| Cervical (Reliability) |  |  |  |
| Brachial plexus tension | Wainner [143] | *k=*0.76-0.81 |  |
| Spurling |  | *k=*0.60-0.88 |  |
|  |  |  |  |
| Lumbar (Reliability) |  |  |  |
| SLR | Hunt [110] | *k=*0.48-0.54 |  |
| SLR | Vroomen [111] | *k=*0.68 |  |
| Bragard’s test | Vroomen [111] | *k=*0.66 |  |
| WLR | Vroomen [111] | *k=*0.70 |  |
| FNST | McCombe [112] | *k=*0.3-0.5 |  |
| Slump test | Philip [113] | *k=*0.72-1.0 |  |
| Slump test | Gabbe [114] | *ICC*=0.92 |  |
|  |  |  |  |
| Cervical (Validity) |  |  |  |
| Test clusters | Wainner [32] |  | Three tests positive:  SE = 0.39  SP = 0.94  PLR = 6.1  Four tests positive:  SE = 0.24  SP = .99  PLR = 30.3 |
| Spurling’s test | Shah [33] |  | SE = 0.90  SP = 1.00  PPV = 1.0  NPV = 0.71 |
|  |  |  |  |
| Lumber (Validity) |  |  |  |
|  | Lurie [117] |  | Systematic Review |
|  | Stankovic [118] |  | Increased likelihood of leg pain on Slump Test with herniated disc (*p* < 0.017) |
|  |  |  |  |
| Muscle Palpation Signs |  |  |  |
| Cervical (Reliability) | Marcus [20] | Cervical spine:  *k=*0.74;  Head: *k* = 0.81;  Shoulder *k*=1.0 |  |
|  | Van Suijlekom [22] | *k*=0.0-1.0 |  |
|  | Gerwin [34] | *S(av)* = 0.0 -1.0 |  |
|  | Sciotti [35] | IER = 0.83-0.92 |  |
|  | Lew [36] | 10-21% agreement |  |
|  |  |  |  |
| Lumbar (Reliability) |  |  |  |
| Trigger point | Nice [119] | *k*=0.29-0.38 |  |
|  | Njoo [120] | *k*>0.5 |  |
|  | Hsieh [144] | *k*= -0.001-0.453 |  |
|  |  |  |  |
| Cervical and Lumbar (Validity) | None |  |  |

*k* = kappa; SE= sensitivity; SP=specificity; PPV=positive predictive value; NPV=negative predictive value; PLR=positive likelihood ratio; -NPV=negative likelihood ratio; ICC=intraclass correlation coefficient; SLR=straight leg raise; WLR=well leg raise; FNST=femoral nerve stretch test.
