# Supplementary material for: A diagnosis-based clinical decision rule for spinal pain part 2: review of the literature
Source: Chiropr Osteopat. 2008 Aug 11;16:7. doi: 10.1186/1746-1340-16-7 (PMC2538525; doi:10.1186/1746-1340-16-7)
Supplement: Additional file 4 — Table 4. Findings from studies related to question 3. [file 1746-1340-16-7-S4.doc]

Table 4. Findings from studies related to question 3.

| Dynamic Instability |  |  |  |
| --- | --- | --- | --- |
| Cervical (Reliability) |  |  |  |
| CF test | Jull [38] | ICC = 0.81 – 0.93 |  |
|  | Chiu [39] | *k*=0.72 |  |
|  | Cleland [23] | ICC = 0.57 |  |
|  | Harris [40] | ICC = 0.67 |  |
|  | Olson [41] | *k*=0.83-0.88 |  |
|  |  |  |  |
| Lumbar (Reliability) |  |  |  |
| Segmental Instability | Hicks [123] | *k*=0.87 |  |
| Hip Extension | Murphy [145] | *k*=0.72-0.76 |  |
| Standing Flexion Test | Hicks [123] | *k*=0.72-0.76 |  |
|  |  |  |  |
| Pelvis (Reliability) |  |  |  |
| ASLR | Mens [126] | Test/retest  ICC – 0.83 |  |
|  |  |  |  |
| Cervical (Validity) |  |  |  |
| CF | Treleaven [26] |  | *p*=0.02 |
| CF | Jull [38] |  | P<0.001 |
| CF | Jull [42] |  | P<0.05 |
| CF | Falla [43] |  | (*p*=0.002) decreased activation of deep cervical flexors in neck pain patients compared to control |
|  |  |  |  |
| Lumbar (Validity) |  |  |  |
| IV motion test | Abbott [127] |  | SE = 0.29 – 0.33  SP = 0.88 – 0.89  PLR = 2.52 – 2.74  NLR = 0.76 – 0.81 |
|  |  |  |  |
| Pelvis (Validity) |  |  |  |
| ASLR | Mens [126] |  | SE = 0.87  SP = 0.94 |
|  | Mens [128] |  | R=0.70 |
|  |  |  |  |
| Central Pain Hypersensitivity |  |  |  |
| Waddell’s signs | Fishbain [45] |  | Systematic review |
| Non-organic signs | Sobel [46] | *k*=0.80-1.0 |  |
|  |  |  |  |
| Oculomotor dysfunction |  |  |  |
|  | Gimse [51] |  | P<0.001 |
|  | Tjell [52] |  | P<0.05-0.0001 |
|  | Heikkilla [53] |  | Significant (p= 0.007) correlation between cervical kinesthetic tests and oculomotor tests |
|  | Revel [54] |  | P<0.01 |
|  | Loudon [55] |  | P<0.05 |
|  | Treleaven [56] |  | SE = 0.60  SP =0.54  PPV = 0.88 |
|  |  |  |  |
| **Fear and Catastrophizing** |  |  |  |
| Tampa Scale for Kinesiophobia | Swinkels-Meewisse [132] |  | Significant (p=0.001 predictor of present pain and disability) |
| Tampa Scale for Kinesiophobia | Swinkels-Meewisse [133] |  | Significant (p<0.001) predictor of risk of pain and disability |
| Tampa Scale for Kinesiophobia | Nederhand [60] |  | Predicting chronicity in whiplash patients LR = +1.7; PPV = 45.5; LR- = 0.16; NPV = 7.7 |
| Fear-Avoidance Beliefs Questionnaire | Woby [134] |  | Significant (p < 0.01 for Activity scale; p < 0.05 for Work scale) predictor of disability |
| Pain Catastrophizing Scale | Severeijns [130] |  | Significant (*p* < 0.01) predictor of pain and disability |
|  |  |  |  |
| Passive coping |  |  |  |
|  |  |  |  |
| Vanderbilt Pain Management Inventory | Carroll [65] |  | Continuous scale score – HRR 0.95  Low passive score - HRR 1.00  Moderate passive score - HRR 0.76  High passive score - HRR 0.43 |
| Chronic Pain Coping Inventory | Truchon [131] |  | Significant (p<0.05) predictor of pain and disability |
| Coping Strategies Questionnaire | Koleck [135] |  | Significant (p<0.05) predictor of disability |
|  |  |  |  |
| **Depression** |  |  |  |
| Mental Component Summary of SF-36 | Walsh [137] |  | SE = 0.80  SP = 0.90 |
| Two question screen | Haggman [140] |  | PLR = 5.4  NLR 0.18 |
| Center for Epidemiologic Studies Depression (CES-D) Scale | Carroll [65] |  | HRR = 0.68 |

*k* = kappa; SE= sensitivity; SP=specificity; PPV=positive predictive value; NPV=negative predictive value; PLR=positive likelihood ratio; -NPV=negative likelihood ratio; ICC=intraclass correlation coefficient; HRR = hazard rate ratio
